# Evaluation of phenotypic and functional stability of RAW 264.7 cell line through serial passages

**DOI:** 10.1371/journal.pone.0198943

**Published:** 2018-06-11

**Authors:** Bartłomiej Taciak, Maciej Białasek, Agata Braniewska, Zuzanna Sas, Paulina Sawicka, Łukasz Kiraga, Tomasz Rygiel, Magdalena Król

**Affiliations:** 1 Department of Physiological Sciences, Faculty of Veterinary Medicine, Warsaw University of Life Sciences, Warsaw, Poland; 2 Department of Immunology, Centre for Biostructure Research, Medical University of Warsaw, Warsaw, Poland; Center for Cancer Research, UNITED STATES

## Abstract

Established cell lines are widely used in research, however an appealing question is the comparability of the cells between various laboratories, their characteristics and stability in time. Problematic is also the cell line misidentification, genetic and phenotypic shift or *Mycoplasma* contamination which are often forgotten in research papers. The monocyte/macrophage-like cell line RAW 264.7 has been one of the most commonly used myeloid cell line for more than 40 years. Despite its phenotypic and functional stability is often discussed in literature or at various scientific discussion panels, their stability during the consecutive passages has not been confirmed in any solid study. So far, only a few functional features of these cells have been studied, for example their ability to differentiate into osteoclasts. Therefore, in the present paper we have investigated the phenotype and functional stability of the RAW 264.7 cell line from passage no. 5 till passage no. 50. We found out that the phenotype (expression of particular macrophage-characteristic genes and surface markers) and functional characteristics (phagocytosis and NO production) of RAW 264.7 cell line remains stable through passages: from passage no. 10 up to passage no. 30. Overall, our results indicated that the RAW 264.7 cell line should not be used after the passage no. 30 otherwise it may influence the data reliability.

## Introduction

Established cell lines are widely used all over the world for both *in vitro* and *in vivo* analyses. The reason for that is unlimited source of cells with similar genotype and phenotype through consecutive passages. However, in recent years authors are more cautious in interpretation of data obtained from experiments conducted only on the established cell lines. The most appealing question is obviously the characteristics of the cells and their comparability and stability between various laboratories and even various passages [1]. Cell line misidentification, *Mycoplasma* contamination or genotypic and phenotypic shift when cultured for prolonged time, are the important issues very often missed in research papers. The immortalized cell lines are usable through many passages but their increasing number of passages enhances probability of genotype and phenotype change [2]. In most of the research papers (even these published in top journals) there is no information about the passage number of the used cell lines or phenotype characteristics. The use of many cell lines has been questioned suggesting superiority of primary cells over the established cell lines [3–6]. Comparison of cells of the lower passages with cells of the higher passages being continuously in culture, demonstrated significant changes in their morphology, growth and differentiation ability with their consecutive passages [7,8]. From this point of view, macrophages are particularly interesting cells. They are famous for their plasticity and possibility to polarization and differentiation to osteoclasts, Kuppfer cells or dendritic cells [9–11]. Therefore, macrophages are a very diverse cell type. They are found in every organ and tissue in the body, however their phenotype is very different depending on the physiological state [12]. Macrophages are very sensitive to environmental conditions and are able to quickly adapt to new stimuli [13]. Depending on the type of activation manifested by produced cytokines and expression of surface markers, macrophages are classified as the M1 or M2 subgroups. The M1 macrophages (iNOS^+^, CD80^+^, MHCII^+^), classically activated (e.g. by LPS or IFN-γ) are pro-inflammatory cells, secreting following cytokines: IFN-γ and TNF-α. On the other hand, anti-inflammatory M2 macrophages (Arg-1^+^_,_ CD163^+^, CD206^+^) are induced by IL-4 or IL-10 [14,15].

The RAW 264.7 cells are monocyte/macrophage-like cells, originating from Abelson leukemia virus transformed cell line derived from BALB/c mice. These cells are being described as an appropriate model of macrophages. They are capable of performing pinocytosis and phagocytosis. Upon LPS stimulation RAW 264.7 cells increase nitric oxide (NO) production and enhance phagocytosis. Furthermore, these cells are able to kill target cells by antibody dependent cytotoxicity [16]. The phenotypic and functional stability of RAW 264.7 macrophages is very often discussed in literature or at various scientific discussion panels (e.g. Research Gate). However, despite RAW 264.7 cell line has been used in biological laboratories for more than 40 years, so far their stability has not yet been confirmed. When RAW 264.7 cells are stimulated by Receptor Activator for Nuclear Factor κ B Ligand, they differentiate into osteoclasts. Osteoclasts differentiated from RAW 264.7 acquire activity of Tartrate-Resistant Acid Phosphatase (TRAP) which is the best know marker of osteoclasts [17]. The osteoclast model has been used for many years, however it is not known at which passage RAW 264.7 lose their ability to such a differentiation [18].

The American Type Culture Collection (ATCC), the main supplier of cell lines recommends their use till the passage no. 18. This recommendation is based on the results of one study showing that RAW 264.7 ability to differentiate into osteoclasts remains until the passage no. 18 [18].

We decided to investigate the phenotype and functional stability of the RAW 264.7 cell line from passage no. 5 till passage no. 50. Studying transcriptional expression data and expression of surface protein markers, we investigated their phenotype, whereas their functional capacity was studied by investigation of their ability to phagocytose and to produce NO.

## Materials and methods

### Cell line

The RAW 264.7 cell lines (third passage) were purchased from American Type Culture Collection (ATCC® TIB-71™) and from Sigma-Aldrich (cat. no. 91062702). Cells were cultured in DMEM high glucose medium supplemented with 10% FBS (all the experiments were performed using the same FBS batch) and 1% penicillin/streptomycin, in atmosphere of 5% CO_2_ and 95% humidity at 37°C. Cells were passaged after reaching 90% confluence, detached with cell scraper and subcultivated in 1:6 ratio in T-75 flasks. All cell culture equipment (flasks, pipettes etc.) used in this study were from the same batch. Cells were cultured continuously from the third passage until passage no. 50. Cells were frozen every fifth passage starting from the passage number three. Before phenotype and functional analysis cells were thawed and cultured for next two passages (e.g. cells of passage number three were thawed and cultured until passage no. 5 and then subjected to further analyses). Cells were regularly tested for *Mycoplasma* contamination. To avoid differences between culture techniques, cells were cultured only by one person.

### Real time PCR

Total RNA from one million of cells suspended in Trizol was isolated on silica columns (A&A Biotechnology) followed by the cDNA synthesis (Roche). Quantitative RT-PCR was performed using a fluorogenic SYBR Green Master Mix kit (Thermo Fisher) and a Mx3005P QPCR System (Agilent). Data were analyzed using the comparative Ct method [19]. Sequences of key genes involved in monocyte/macrophage biology were obtained from Primer Bank [20] (S1 Table). Ten different genes were tested as possible housekeeping genes. Finally, results were normalized to eukaryotic elongation factor 2 (*eef2*) housekeeping gene. Expression of other genes existing in the literature as possible housekeeping genes (Actb, B2m, Gusb, Hprt, Hsp90ab1, Ldha16b, Nono, Ppia, Rpl13a and Tbp) was less stable (S1 Fig). Gene expression was analyzed in RAW 264.7 cells of the following passage no.: 5, 10, 15, 20, 30 and 50. This analysis has been performed on the cells obtained from two various cell sources in triplicate.

### Measurement of surface markers expression level

Monoclonal antibodies for mouse CD11b, CD11c, CD86, CD200R, CXCR4 and Ly6C were obtained from eBiosciences. The RAW 264.7 cells were blocked using 5% rat serum and stained with antibodies according to manufacturer`s recommendation. Cells were analyzed by BD FACS Aria II (Becton Dickinson). Flow cytometry data were analyzed using FlowJo software. This analysis has been performed on the cells obtained from two various cell sources in triplicate.

### Phagocytosis assay

Phagocytosis assays were performed with Phagocytosis Assay Kit (Cayman Chemicals) according to manufacturer`s protocol. The RAW 264.7 cells were plated on 24-well plates for 24 hrs. Control cells were grown in standard culture conditions, however activated cells were treated with 100 ng/ml LPS (Sigma-Aldrich) for 2 hrs. To evaluate their phagocyte activity, FITC conjugated latex beads were administered for 2 hrs followed by the analysis using flow cytometry (BD FACS Aria II) and confocal microscopy FV-500 system (Olympus Optical Co.). As a negative control were used cells treated as above but at the temperature 0°C. This analysis has been performed on the cells obtained from two various cell sources in triplicate.

### NO production assay

The RAW 264.7 cells were cultured at 96-well plates for 24 hrs (1.5x10^5^ cells/well) in normal conditions or in normal growth medium supplemented with 100 ng/ml of LPS. Culture supernatants (equal volumes) were mixed with Greiss reagent (G4410; Sigma-Aldrich) and then the absorbance was measured at the wavelength 570 nm using a microplate reader (Infinite f200, TECAN), as described previously [21]. This analysis has been performed on the cells obtained from two various cell sources in triplicate.

### Statistical analyses

Data are presented as mean ± standard deviation (SD). The one way variance analysis ANOVA and HSD Tukey post-hoc test were used for the comparison of means between cells from different passages. Statistical analyses were performed using the GraphPad Prism software (GraphPad Software, San Diego, CA), *p* value < 0.05 was considered as significant, whereas *p* value < 0.01 was considered as highly significant.

## Results and discussion

The RAW 264.7 cells are often used for *in vitro* and *in vivo* studies as an appropriate monocyte/macrophage model [22]. However, their stability between various laboratories and passages is questionable [1]. The ATCC, the main supplier of the cell lines, recommends their use till passage no. 18, however without showing any strong rationale for this [18].

The usage of immortalized cell lines through many passages increases probability of their genotype and phenotype changes [2]. In literature there is a lot of information about the variability of different cell lines between passages. Older cells usually show changes in their morphology, ability to grow and differentiate, they produce less proteins and are more resistant to transduction [8]. However, there is no rule regarding classification criteria of cell lines as younger or older and it seems to be cell line dependent. For example expression of mRNAs involved in secretion, adhesion and proliferation in MIN-6 pancreatic carcinoma at passage no. 18 differs significantly from those at passage no. 40 [23]. Lin et al. showed different regulation of sex hormone receptors by PI3K/Akt signaling pathway in prostate cancer cells (LNCaP) at passage no. 25 and no. 60 [24]. However, in most of research papers (even these published in top journals) usually authors do not provide the information about the passage number of the used cell line.

The question regarding phenotypic stability of RAW 264.7 cell line is quite often raised up, however there is still no conclusion about that. The only publication addressing this problem was published in 2012 by Collin-Osdoby et al. The authors recommended use of RAW 264.7 cell line up to passage no. 18, due to their later decrease in ability to differentiate into osteoclasts [18]. Therefore, confirmation of RAW 264.7 stability through various passage numbers is crucial for the proper further data interpretation.

**Fig 1:**
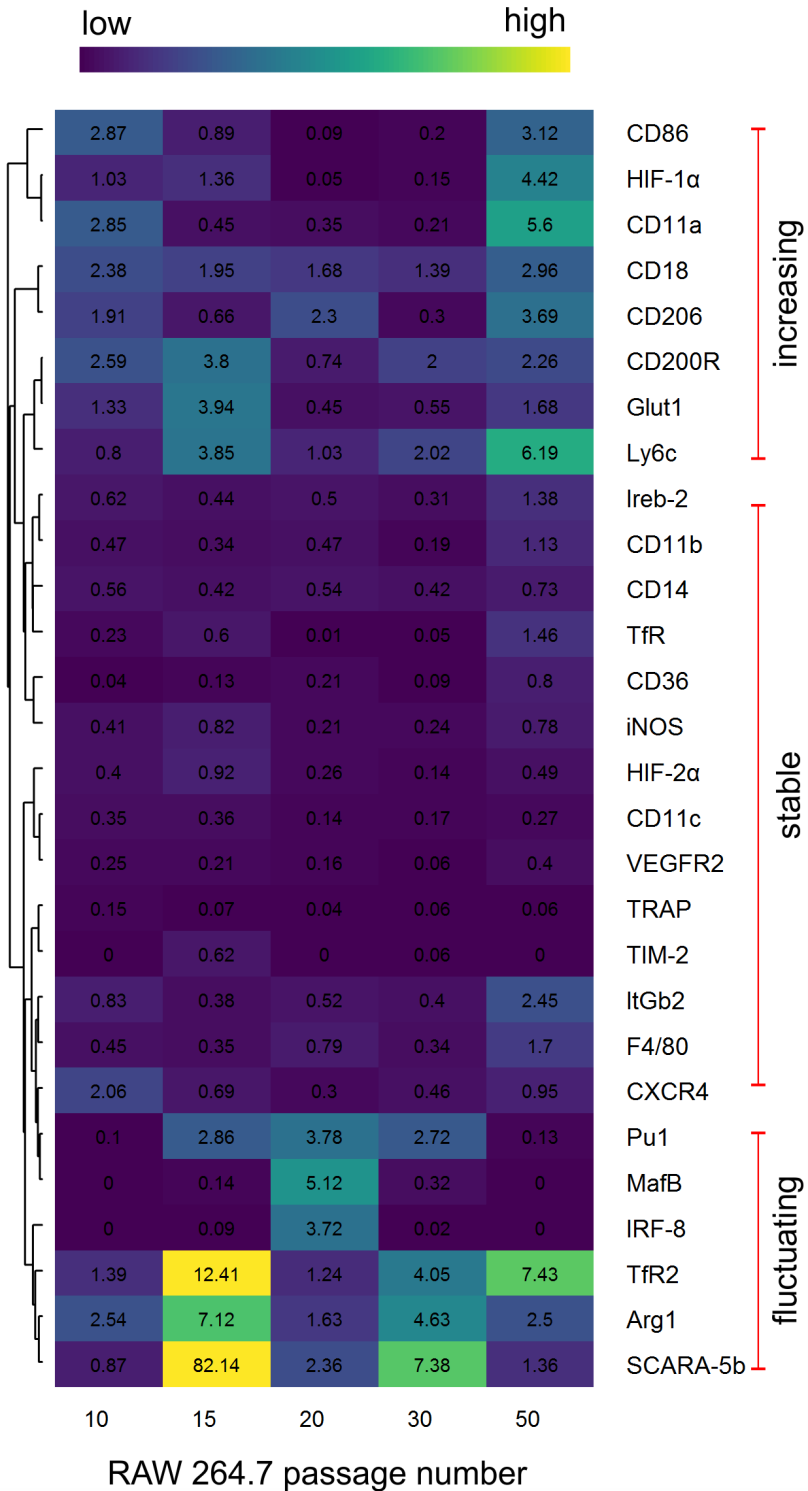
qPCR analysis. Heat map showing qPCR analysis results of selected genes in various passages of RAW264.7 cells. Differences in gene expression are represented by ΔΔCt values of three biological and three technical replicates. The control for this experiment constituted cells from the passage no. 5. Their gene expression value equals 1. Analysis was performed using R (https://cran.r-project.org) package gplots [25].

To clarify the phenotype stability of RAW 264.7 macrophages we performed analyses of their gene expression (we selected the panel of 28 genes involved in macrophage metabolism) and their surface markers expression. Based on our results and statistical analysis the expression of genes in RAW 264.7 from passage no. 5 to passage no. 50 can be allocated to the three expression pattern groups. We described these groups as: increasing expression (through passages), stable expression and fluctuating expression (Fig 1).

In the first group were the genes with stable expression. These genes are involved in macrophage functions: *CD11b*, *CD14*, *Ireb-2*, *TfR*, *CD36*, *iNOS*, *CD11c*, *VEGFR2*, *TRAP*, *TIM-2* and *HIF-2α*. Expression of all the genes from this panel was not significantly changed from passage no. 5 up to passage no. 50 (Fig 1). Whereas, expression of *ItGb2* and *F4/80* was significantly increased in the passage no. 50 as compared to initial passage no. 5. As it is showed on the Fig 1, for all of these genes the ΔΔCt value in almost every studied passage was smaller than 1. It means that gene expression was down-regulated in comparison to passage no. 5.

Another set of genes, also associated with macrophage activation: *CD86*, *HIF-1α*, *CD11a*, *CD18*, *CD206*, *CD200R*, *Glut1* (Glucose transporter 1) and *Ly6c* showed increased expression through consecutive passages: from passage no. 5 to passage no. 50.

Third set of genes: *TfR2* (transferrin receptor 2), *Arg1* and *SCARA-5b* (scavenger receptor class A member 5) showed the highest expression in older passages, that is after no. 15 and no. 20. Very similar expression pattern was observed in case of main macrophage transcription factors: *Pu1*, *MafB* and *IRF-8*. However, the differences between various passages were smaller. The highest expression was noted in passage no. 20. Importantly, no significant differences between passages no. 5, 10, 15, 30 and 50 in expression of *MafB* and *IRF-8* transcription factors were observed. The *Pu1* expression was higher in passages no. 15 and no. 30 than in other passages.

Our study showed that there are significant differences between gene expression in RAW 264.7 cells at various number of passages. In case of genes responsible for cell adhesion *CD36* and *CD11b* [26] we observed different expression between early passage no. 5 and every older passage. Similarly, the expression of *cd11b* was significantly decreased in older passages. Expression of *F4/80* regulates not only cell adhesion, but also induction of CD8+ lymphocyte, and it remained stable up to passage no. 50.

It is worth to mention that there is a set of genes (*Ireb-2*, *CD11b*, *CD14*, *TfR*, *CD36*, *iNOS*, *HIF-2α*, *CD11c*, *VEGFR2*, *TRAP*, *TIM-2*, *ItGb2*, *F4/80*) which expression after passage no. 5 subsequently decreased till passage no. 50 when it increased. However, between passages no. 10 and 50 their expression was stable.

We also observed differences in expression of genes responsible for phagocytosis (*Cd206*, *Itgb2*, *Scara-5b*) in cells at various passages. Just in case of one gene: *Scara-5b*, cells at passage no. 15–30 showed its significantly higher expression. Expression of *Trap*, involved in ROS production catalysis during phagocytosis remained stable in cells older than at passage no. 5.

For macrophage biology particularly important are the following genes: *Arg1*, *Cd200R* and *iNOS*. The expression of genes inhibiting iNOS and reducing inflammation: *Arg1* and *Cd200R* was increased after passage no. 5 but differences in expression between various passages were not significant. The only exemption was *Arg1* in passage no. 15 when expression was significantly higher. Accordingly, in a line with these results, *iNOS* expression was more stable and significantly lower than expression of these two, mentioned before, genes. These genes are functionally correlated with other genes, involved in iron metabolism: *TfR*, *TfR2 and Tim-2*. Besides *TfR* whose expression dropped down in cells at passage no. 10–30 the expression of other genes remained stable through various passage numbers.

Among the genes involved in angiogenesis (*CxcR4*, *Hif-1α*, *Hif-2α*, *VegfR2*) all of them showed significant differences in early passages (from no. 5 to 15) where expression was higher in comparison to older passages, excluding *Hif-1α* with high expression in passage no. 50.

Expression of genes involved in T cell activation and glucose transport *Cd86* and *Glut 1* remained stable between various numbers of passage of RAW 264.7 cells until passage no. 50 where expression was higher.

The increased or decreased expression of many studied genes is correlated with expression of main macrophage transcription factors: *Pu1*, *MafB* and *IRF-8*. The *MafB* and *IRF-8* expressions remained low until passage no. 15. The *Pu-1* remained stable until passage no. 10. After these mentioned passage numbers, the expression started to subsequently increase. In case of *MafB* and *IRF-8* it increased till passage no. 20, for *Pu1* till passage no. 30 and then decreased.

Based on the above data of gene expression we conclude that cells of RAW264.7 cell line remain stable until passage no. 30 but for many genes (*TfR2*, *Arg1*, *Scara-5b*, *Ly6c*, *Glut1*, *CD200R*, *CD18*, *Pu1*, *MafB*, *IRF-8*) the crucial changes happened between passages no. 15–20.

We are aware that it is extremely difficult to extract RNA and proteins from cells from various passages under exactly the same conditions. In particular, it is difficult to accurately adjust the confluence of cells in each experiment. In order to limit possibility of human error, the manipulations with cells, especially cell culture were performed by one person. The cells were purchased from two different sources and RNA for analysis was isolated from a few different cultures (6 technical culture flasks, three for every biological source of cells). Doing that, we limited the influence of various conditions, e.g. confluence and medium condition on the gene expression. Additionally we used one batch of FBS for the whole experiment.

**Fig 2:**
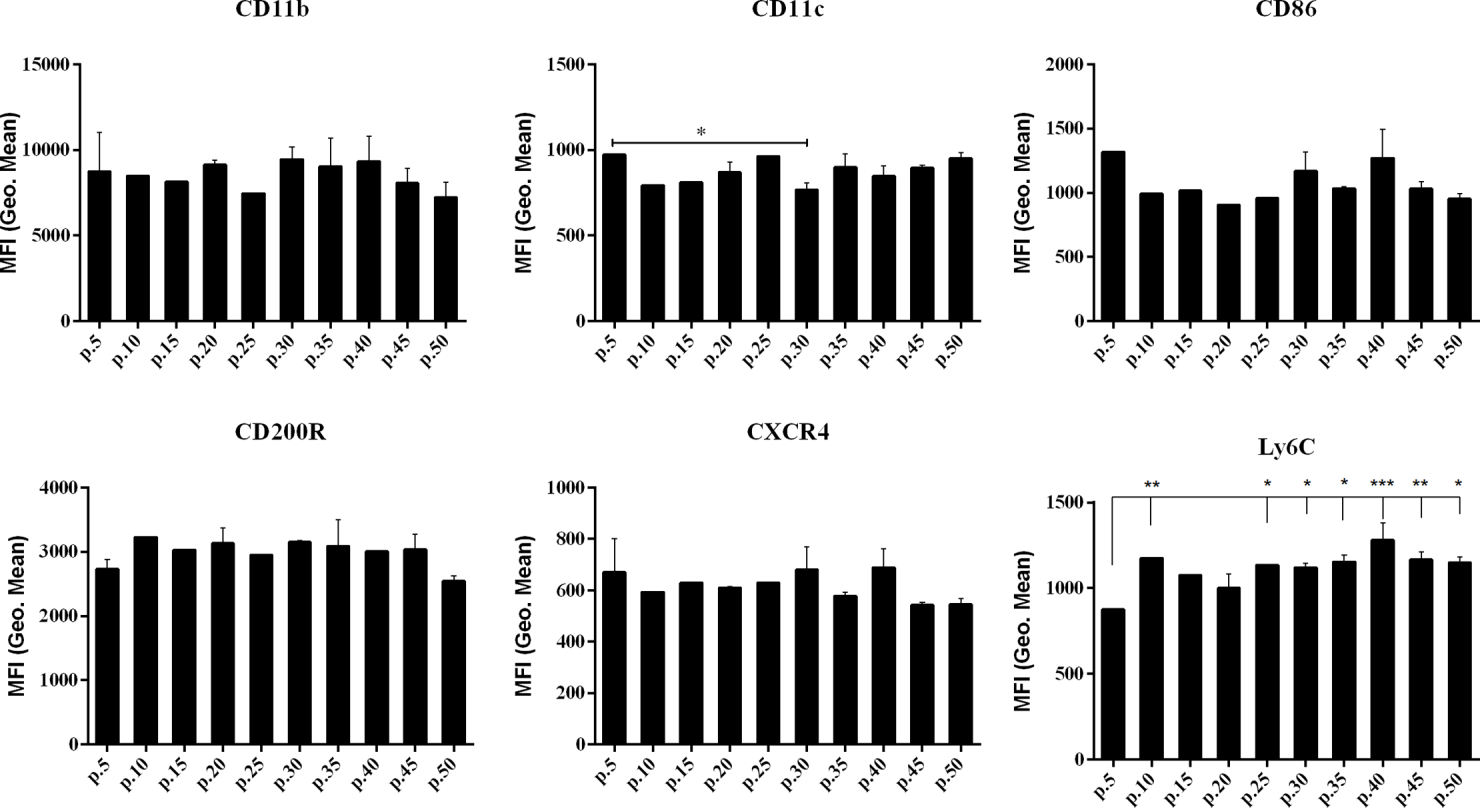
Flow cytometry analysis. Flow cytometry analysis of CD11b, CD11c, CD86, CD200R, CXCR4 and Ly6C surface markers expression on RAW 264.7 cells. Values of expression are expressed as geometric mean of MFI (Mean Fluorescence Intensity). *- p-value < 0.05, **—p-value < 0.01.

In order to investigate stability of the RAW 264.7 biological characteristics, we evaluated expression of macrophage markers at protein level. The RAW 264.7 cells of the following passages no.: 5, 10, 15, 20, 25, 30, 35, 40, 45 and 50 were stained with antibodies against CD11b, CD11c, CD86, CD200R, CXCR4 and Ly6C antigens. Expression analysis of these markers was performed by flow cytometry (Fig 2). Cell surface expression of CD11b, CXCR4, CD86 and CD200R remained stable in cells through all the examined passages. Cells at passage no. 30 showed decreased expression of CD11c comparing with other cell passages, especially with passage no. 5. We have also observed difference in Ly6C expression at passage no. 5 comparing with older cells. The increased expression of surface marker Ly6C on protein level confirmed the transcriptional data, where a gradual increase in mRNA expression was also measured (Fig 1). Other macrophage surface markers remained stable at various passages showing their high phenotypic stability.

Because macrophages show high plasticity, their functional analysis is even more important than their phenotype analysis. Therefore, we analyzed their ability to perform two of the most important macrophage processes: phagocytosis and nitrogen oxide production.

**Fig 3:**
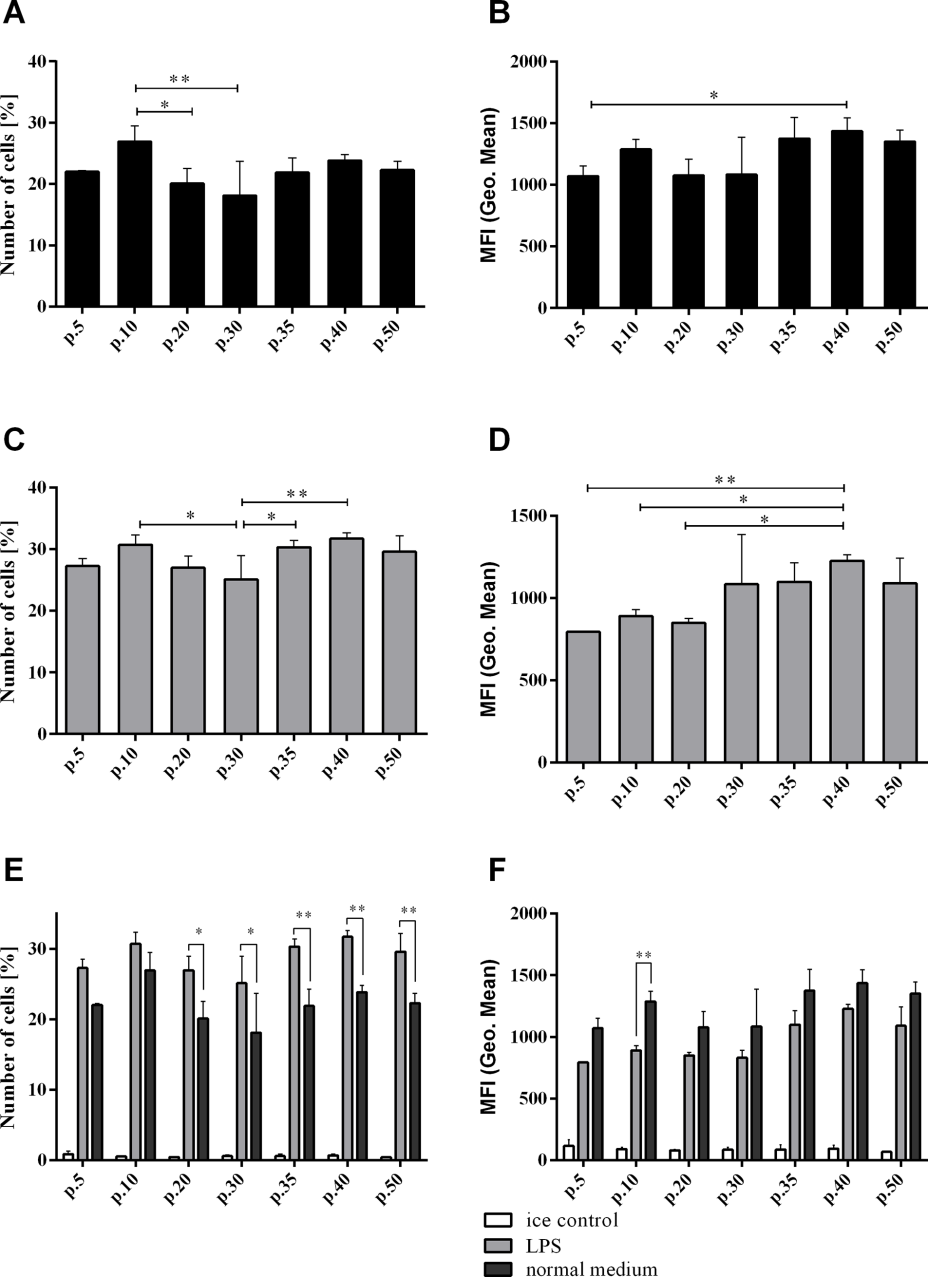
Phagocytosis assay. Results of phagocytosis assay performed on RAW 264.7 cells at various passages. Graphs A, C, E show percentage of phagocyting cells, positive for FITC fluorochrome conjugated with latex beads. Graphs B, D, F show the intensity of phagocytosis expressed as mean fluorescence intensity of latex beads. A, B–phagocytosis of RAW 264.7 cells. C, D–phagocytosis of LPS treated RAW 264.7 cells. E, F–comparison of phagocytosis ability of RAW 264.7 cells in various condition, ice control indicating technical control for phagocytosis assay. *- p-value < 0.05, **—p-value < 0.01.

The essential function of macrophages is the capacity to phagocytose. Because it is commonly known that LPS increases macrophage ability to phagocytose [27], we performed phagocytosis assay on unstimulated cells, and additionally on LPS-treated cells from the following passages no.: 5, 10, 20, 30, 35, 40 and 50. Approximately 20% of unstimulated cells showed phagocytosis activity (Fig 3A). Treatment with LPS increased the number of phagocyting cells to 30% (Fig 3C). However, the level of phagocytosis (measured as mean fluorescence intensity) decreased in every next passage (Fig 3B and 3D), which can be related with LPS concentration and treatment time [28]. The increase of phagocytic cells after LPS treatment is associated with Ly6C expression (Fig 2), as Ly6C protein is responsible for LPS response and phagocytosis regulation [26]. The magnitude of RAW 264.7 cells response to LPS treatment differed at various passages. We did observe differences in RAW 264.7 cells ability to perform phagocytosis between various numbers of cell passages. The differences were in line with gene and protein expression data. The most significant phagocytosis difference between passages was observed between passage no. 10 and passage no. 30, especially after LPS treatment (Fig 3C and 3D). Expression of genes responsible for phagocytosis (*Scara-5b*) was also significantly higher in cells at passage no. 10–30 compared with other passages. In the same cell passages (no. 15–30), we also observed an increased expression of transcription factors (e.g. *Pu1*). The *Pu1* transcription factor is involved in regulation of phagocytosis-related genes and higher ability to phagocytose. Its low expression in the cells of passage no. 5 is in line with their lower ability to phagocytose (LPS-activated or unactivated).

**Fig 4:**
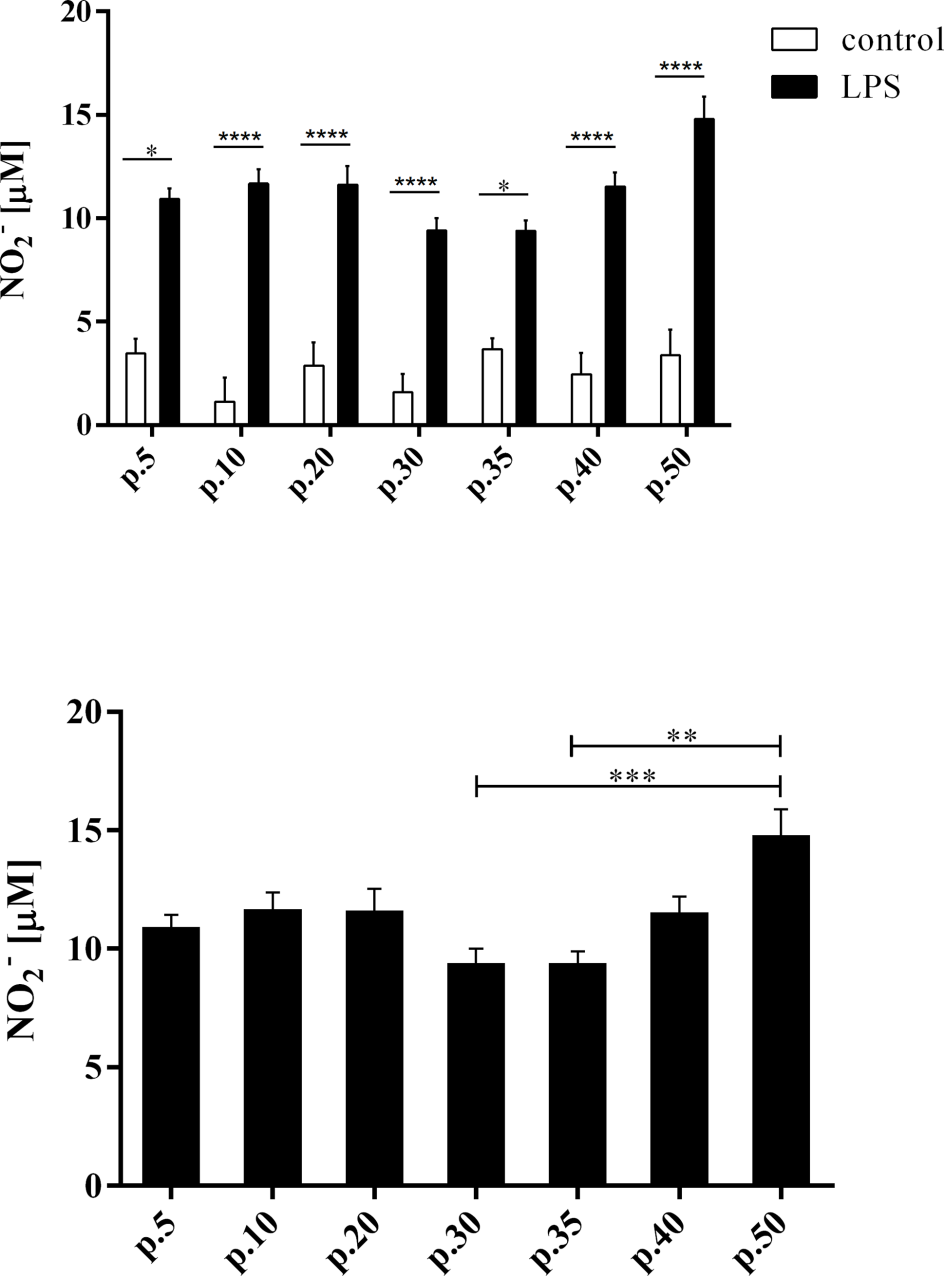
Nitrogen level in cell culture medium. Level of nitrogen in cell culture medium of RAW 264.7 cells at various passages. A—comparison of NO level in control cells and LPS stimulated cells. B– 24 hrs production of nitric oxide by LPS stimulated RAW 264.7 cells. Data presented as average concentration of produced nitrites, calculated on the standard curve basis. *—p-value < 0.05, **—p-value < 0.01, ***—p-value < 0.001, ****—p-value < 0.0001.

In order to study the cytotoxic properties of RAW 264.7 macrophages at various passages, we examined their ability to produce NO in control conditions and after LPS stimulation. This is important function of macrophages during inflammation [29]. As expected, ability of RAW 264.7 cells to produce NO was significantly higher in response to LPS-activation (Fig 4). However, the level of NO production was similar in the cells at various passages. In general we showed significant decrease in NO production in cells at the passages no. 30 and no. 35 and increase in cells at the passage no. 50.

We conclude that the phenotype (expression of particular macrophage-characteristic genes and surface markers) and functional characteristics (phagocytosis and NO production) of RAW 264.7 cell line remains stable through passages: from passage no. 10 up to passage no. 30. The gene expression of RAW 264.7 cells was the most stable between passages no. 10 and 30. In this passage-interval we showed significant increase in expression of three transcription factors essential for macrophages. These results are supported by the finding obtained with functional assays: phagocytosis and NO production assays. Although the expression of several genes seem to be increased at passage no.50, functional studies indicated that the ability of phagocytosis and NO production remains stable through passages up to passage no. 50. However, we did not study all the macrophage functions, that may be influenced by the gene expression changes. Our results are also consistent with ability of RAW 264.7 to differentiate into osteoclasts only before the passage no. 20. Overall, our results indicated that the RAW 264.7 cell line should not be used after the passage no. 30 otherwise it may influence the data reliability.

## Supporting information

S1 Fig Expression of housekeeping genes in RAW 264.7 cells at passages no. 5, 10, 15, 20 and 30 (p.5, p.10, p.15, p.20 and p.30). The values of expression are presented as 1/Ct. *- p-value < 0.05, **—p-value < 0.01, ***—p-value < 0.001.
(TIF)

S1 Table Sequences of primers used in PCR reactions. (DOC)
