# Supplementary material for: Evaluation of phenotypic and functional stability of RAW 264.7 cell line through serial passages
Source: PLoS One. 2018 Jun 11;13(6):e0198943. doi: 10.1371/journal.pone.0198943 (PMC5995401; doi:10.1371/journal.pone.0198943)
Supplement: S1 Table — (DOC) [file pone.0198943.s002.doc]

**Supplementary Table 1 –** Sequences of primers used in PCR reactions.

| Gene | NCBI gene ID | Forward Primer | Reverse Primer | Amplicon Size |
| --- | --- | --- | --- | --- |
| CD11b | 16409 | ATGGACGCTGATGGCAATACC | TCCCCATTCACGTCTCCCA | 203 |
| CD11c | 16411 | CTGGATAGCCTTTCTTCTGCTG | GCACACTGTGTCCGAACTCA | 113 |
| CD14 | 12475 | CTCTGTCCTTAAAGCGGCTTAC | GTTGCGGAGGTTCAAGATGTT | 191 |
| CD86 | 12524 | TGTTTCCGTGGAGACGCAAG | TTGAGCCTTTGTAAATGGGCA | 70 |
| CD206 | 17533 | CTCTGTTCAGCTATTGGACGC | CGGAATTTCTGGGATTCAGCTTC | 132 |
| TfR | 22042 | GTTTCTGCCAGCCCCTTATTAT | GCAAGGAAAGGATATGCAGCA | 152 |
| Ireb-2 | 64602 | TTCTGCCTTACTCAATACGGGT | AGGGCACTTCAACATTGCTCT | 129 |
| F4/80 | 13733 | TGACTCACCTTGTGGTCCTAA | CTTCCCAGAATCCAGTCTTTCC | 111 |
| CD200R | 57781 | TAAGGTGGAGGCATTTCCAGT | GATTCCAATGGCCGACAAAGTA | 76 |
| Ly6C | 17067 | GCAGTGCTACGAGTGCTATGG | ACTGACGGGTCTTTAGTTTCCTT | 140 |
| TIM-2 | 171284 | AGGTGCCGTGGAGTCTCATA | CCCCAACACATAGGAACGATTC | 108 |
| SCARA-5b | 71145 | CATGGATTTCACAATGATTCGCC | TCCCCGTCCTTCTTGTCCC | 123 |
| Arg1 | 11846 | CTCCAAGCCAAAGTCCTTAGAG | AGGAGCTGTCATTAGGGACATC | 185 |
| iNOS | 18126 | GTTCTCAGCCCAACAATACAAGA | GTGGACGGGTCGATGTCAC | 127 |
| TRAP | 13866 | CACTCCCACCCTGAGATTTGT | CATCGTCTGCACGGTTCTG | 118 |
| CD11a | 16408 | CCAGACTTTTGCTACTGGGAC | GCTTGTTCGGCAGTGATAGAG | 197 |
| CD18 | 16414 | CAGGAATGCACCAAGTACAAAGT | CCTGGTCCAGTGAAGTTCAGC | 98 |
| 18S rRNA | 19791 | GAATCGAACCCTGATTCCCCGTC | CGGCGACGACCCATTCGAAC | 99 |
| Glut1 | 20525 | CAGTTCGGCTATAACACTGGTG | GCCCCCGACAGAGAAGATG | 156 |
| VEGFR2 | 22339 | TTTGGCAAATACAACCCTTCAGA | GCAGAAGATACTGTCACCACC | 133 |
| CXCR4 | 12767 | GAAGTGGGGTCTGGAGACTAT | TTGCCGACTATGCCAGTCAAG | 125 |
| HIF-1α | 15251 | CCACAGGACAGTACAGGATG | TCAAGTCGTGCTGAATAATACC | 148 |
| HIF-2α | 13819 | CTGAGGAAGGAGAAATCCCGT | TGTGTCCGAAGGAAGCTGATG | 161 |
| CD36 | 12491 | ATGGGCTGTGATCGGAACTG | GTCTTCCCAATAAGCATGTCTCC | 110 |
| ItGb2 | 16414 | CTTTCCGAGAGCAACATCCAGC | GTTGCTGGAGTCGTCAGACAGT | 185 |
| Actb | 11461 | GGCTGTATTCCCCTCCATCG | CCAGTTGGTAACAATGCCATGT | 154 |
| B2m | 12010 | TTCTGGTGCTTGTCTCACTGA | CAGTATGTTCGGCTTCCCATTC | 104 |
| Gusb | 110006 | GGCTGGTGACCTACTGGATTT | GGCACTGGGAACCTGAAGT | 131 |
| Hprt | 15452 | TCAGTCAACGGGGGACATAAA | GGGGCTGTACTGCTTAACCAG | 142 |
| Hsp90ab1 | 15516 | GTCCGCCGTGTGTTCATCAT | GCACTTCTTGACGATGTTCTTGC | 168 |
| Ldhal6b | 106557 | GGGCTACAAGCATCTTGAGAG | GACACGTTGCACCTGACTG | 101 |
| Nono | 53610 | ACGAACCCTAGCGGAAATTGC | AGGTTGCGGACTGTAAGGGAT | 111 |
| Ppia | 268373 | GAGCTGTTTGCAGACAAAGTTC | CCCTGGCACATGAATCCTGG | 125 |
| Rpl13a | 22121 | AGCCTACCAGAAAGTTTGCTTAC | GCTTCTTCTTCCGATAGTGCATC | 129 |
| Tbp | 21374 | AGAACAATCCAGACTAGCAGCA | GGGAACTTCACATCACAGCTC | 120 |
| Eef2 | 13629 | TGTCAGTCATCGCCCATGTG | CATCCTTGCGAGTGTCAGTGA | 123 |
